# BK Virus Replication in the Glomerular Vascular Unit: Implications for BK Virus Associated Nephropathy

**DOI:** 10.3390/v11070583

**Published:** 2019-06-27

**Authors:** Waldemar Popik, Atanu K. Khatua, Noyna F. Fabre, James E. K. Hildreth, Donald J. Alcendor

**Affiliations:** 1 Department of Internal Medicine, Meharry Medical College, Nashville, TN 37208-3599, USA; 2 Department of Microbiology, Immunology and Physiology, Center for AIDS Health Disparities Research, 1005 Dr. D.B. Todd Jr. Blvd., Nashville, TN 37208-3599, USA; 3 Department of Obstetrics and Gynecology, Meharry Medical College, School of Medicine, 1005 Dr. D.B. Todd Jr. Blvd., Nashville, TN 37208-3599, USA

**Keywords:** BK virus, kidney, renal, podocyte, inflammation, cytokines

## Abstract

Background: BK polyomavirus (BKV) reactivates from latency after immunosuppression in renal transplant patients, resulting in BKV-associated nephropathy (BKVAN). BKVAN has emerged as an important cause of graft dysfunction and graft loss among transplant patients. BKV infection in kidney transplant patients has increased over recent decades which correlates with the use of more potent immunosuppressive therapies. BKV infection of the Glomerular Vascular Unit (GVU) consisting of podocytes, mesangial cells, and glomerular endothelial cells could lead to glomerular inflammation and contribute to renal fibrosis. The effects of BKV on GVU infectivity have not been reported. methods: We infected GVU cells with the Dunlop strain of BKV. Viral infectivity was analyzed by microscopy, immunofluorescence, Western blot analysis, and quantitative RT-PCR (qRT-PCR). The expression of specific proinflammatory cytokines induced by BKV was analyzed by qRT-PCR. Results: BKV infection of podocytes, mesangial cells, and glomerular endothelial cells was confirmed by qRT-PCR and positive staining with antibodies to the BKV VP1 major capsid protein, or the SV40 Large T-Antigen. The increased transcriptional expression of interferon gamma-induced protein 10 (CXCL10/IP-10) and interferon beta (IFNβ) was detected in podocytes and mesangial cells at 96 h post-infection. conclusions: All cellular components of the GVU are permissive for BKV replication. Cytopathic effects induced by BKV in podocytes and glomerular endothelial cells and the expression of CXCL10 and IFNβ genes by podocytes and mesangial cells may together contribute to glomerular inflammation and cytopathology in BKVAN.

## 1. Introduction

BK polyomavirus (BKPyV) or BKV is a member of the polyomaviridae family which includes the JC- polyomavirus virus (JCPyV) or JCV, and simian-virus 40 (SV40 virus) [1,2,3,4]. BKV is a small non-enveloped, circular doubled-stranded DNA virus with a genome size of approximately 5kb (kilobases) [2,5]. BKV infection is ubiquitous with subclinical infections of 80–90% in the general population worldwide, but is often associated with pathology in immunocompromised individuals [6,7,8,9]. BKV is also known to cause some forms of encephalitis in HIV-infected patients [10,11]. Primary infection is usually asymptomatic and occurs early in life, with a seroprevalence of 65%–90% in children 5 to 9 years of age and may be transmitted via respiratory and uro-oral and feco-oral borne routes [3,5]. After primary infection, initial viral replication is followed by latency in renal tissue and other anatomical sites [12]. Clinical presentations of BKV infections linked to immunosuppression include diseases of the respiratory tract, urinary bladder, kidney, central nervous system, eye, digestive tract, and endothelium [5]. BKV asymptomatic infection can be accompanied by non-pathogenic transitory viremias, which will remain latent as long as the individual remains immunocompetent; however, BKV reactivates from latency under conditions of immunosuppression [12,13]. BKV reactivation from latency is followed by viral shedding in urine, which occurs in 0–20% of asymptomatic immunocompetent individuals and in 20–60% of immunocompromised patients [14]. Virus reactivation has been strongly associated with the more potent iatrogenic immunosuppressants, such as tacrolimus and mycophenolate, after transplantation [15,16]. This may result in BKV-associated nephropathy (BKVAN), leading to ureteral stenosis, tubular interstitial damage, and hemorrhagic cystitis in bone marrow transplant patients [17]. Approximately 80% of renal transplant recipients will have BK viruria, and 5–10% of those will go on to develop BKVAN [7].

Approximately 50–80% patients who develop BKVAN, will also develop graft failure, depending on the degree of glomerular inflammation cause by proinflammatory cytokines, such as CXCL10, destruction of renal tubular epithelial cells, and the presence of renal fibrosis [18,19]. End-stage renal disease (ESRD) represents an important health disparity among underserved minority populations [20,21,22,23]. Currently, there is no specific treatment for BKVAN. Primary infection and dysfunction of glomerular vascular unit (GVU) cells of the human kidney, consisting of podocytes, mesangial cells, and glomerular endothelial cells could lead to progressive inflammation, injury, and cytolysis of glomerular parenchymal cells and renal fibrosis, and would likely contribute to ESRD [24,25]. The effects of BKV on GVU infectivity has not been reported. The current study examines primary GVU cells of the human kidney for BKV infectivity, cytokine expression profiles post exposure, and the implications for BKVAN.

## 2. Materials and Methods

### 2.1. Cells

Primary human mesangial cells, glomerular endothelial cells, and primary human renal proximal tubular epithelial cells (HRPTEC) were obtained from ScienCell Research Laboratories (Carlsbad, CA) and cultivated in mesangial, endothelial, and epithelial cell media, respectively, from ScienCell. Mesangial cells, glomerular endothelial cells, and HRPTEC were used at passage 3. Immortalized human glomerular podocytes AB8/13 were obtained from Moin A. Saleem (University of Bristol, UK) [26] and were cultured as described [27]. Podocytes were cultured in RPMI media supplemented with 10% FCS and insulin-transferrin-selenium (ITS; ThermoFisher Scientific, Waltham, MA). All cells were plated on uncoated 4.2 cm^2^/well glass chamber slides or 6-well dishes at densities of 2.5 × 10^5^ cells per well and 3.0 × 10^5^ cells per well, respectively. 

### 2.2. BK Virus Preparation, Cultivation, and Titration

BKV VR837 strain, acquired from American Type Culture Collection (Manassas, VA, USA) and used in this study, was originally isolated from urine of a kidney transplant patient in London in 1970 and is referred as the Dunlop strain (nucleotide GenBank: V01108.1, BKV strain, complete genome, [28]). The virus was cultivated in HRPTEC and stock viral titers were performed by florescent focus assays on HRPTEC using the SV40 Large-T antigen antibody from Abcam (Cambridge, MA, USA) [24]. The infectious supernatant was filtered using a 0.22 µm filter and the serum content adjusted to 15%. The infection inoculum was adjusted to approximately 1 × 10^5^ particles per 25 μL of infectious culture supernatant. Heat-killed BKV was prepared by heating the viral inoculum to 70 °C for 30 min in a water bath [29]. All experiments were carried out under biosafety level 2 containment, as recommended. The use of BKV was approved by the Meharry Medical College Institutional Review Board and the Institutional Biosafety Committee. 

### 2.3. BKV RNA Analysis

Total cellular RNA was isolated from the cells using Quick RNA MiniPrep kit (Zymo Research, Irvine, CA, USA), and 500 ng RNA was reverse transcribed into cDNA using iScript cDNA synthesis kit (Bio-Rad Laboratories, Hercules, CA, USA). Real-time PCR was performed on a CFX96 PCR machine (Bio-Rad) using SYBR Green PCR master mix (Bio-Rad) and BKV-specific primers (forward primer 5′-TTTGGACCCACCATTGCA-3′ and reverse primer 5′-AGAGCCCTTGGTTTGGATAGATT-3′) and GAPDH specific primers (forward 5′-GAAGGTGAAGGTCGGAGT-3′ and reverse 5′-GAAGATGGTGATGGGATTTC-3′). The following amplification conditions were used: 95 °C for 3 min for initial denaturation and 40 cycles of 95 °C for 10 s and 60 °C for 45 s. Samples were analyzed in triplicate, and BKV RNA expression was normalized to GAPDH mRNA levels. Data are presented as mean ± standard deviation (SD).

### 2.4. qRT-PCR Analysis of the Proinflammatory Cytokine Gene Expression

Total cellular RNA was isolated, processed, and analyzed as described above. The primers used to analyze cytokine gene expression were as follows: IFNβ: forward 5′-CTTGGATTCCTACAAAGAAGCAGC-3′, reverse 5′-TCCTCCTTCTGGAAC TGCTGCA-3′; CXCL10: forward 5′-TGGCATTCAAGGAGTACCTC-3′, reverse 5′-TTGTAGCAATGATCTCAACACG-3′. Samples were analyzed in triplicate, and cytokine gene expression was normalized to GAPDH mRNA levels, as previously reported [24]. 

### 2.5. Immunofluorescence

Immunofluorescent staining was performed as previously described [Alcendor 2017] in chamber slide cultures containing mock and BKV-infected GVU cells (podocytes, mesangial cells, and glomerular endothelial cells). Briefly, cells were washed twice with PBS, pH 7.4, air- dried, and fixed in absolute methanol for 20 min at −20 °C. Next, cells were air-dried for 10 min, hydrated in Tris-buffered saline (TBS) (pH 7.6) for 10 minutes, and incubated separately for 1 h with monoclonal antibodies to the BKV major capsid protein VP1 (Santa Cruz Biotech, Temecula, CA, USA), von Willebrand factor (Santa Cruz Biotech), nephrin (Santa Cruz Biotech), and SV40 Large T-Antigen (Abcam), all at a dilution 1:50 in PBS pH 7.4 [30].

### 2.6. Western Blot Analysis

Cell extracts were prepared using RIPA lysis buffer [50 mM Tris pH 7.5, 150 mM NaCl, 2 mM ethylenediaminetetraacetic acid (EDTA) pH 8.0, 1% NP40, 0.5% sodium deoxycholate, 0.1% sodium dodecyl sulfate (SDS), and proteinase inhibitor (cOmplete ULTRA, Roche, Basel, Switzerland). Lysates were incubated on ice for 30 minutes and then clarified by centrifugation. Total protein was measured using a micro BCA protein assay kit (ThermoFisher Scientific). Protein lysates (30 μg) were separated by 10% SDS-PAGE, transferred to nitrocellulose membranes (Bio-Rad), blocked with 5% milk in 0.1% TBST (0.1% Tween 20, 20 mM Tris, 150 mM NaCl), and incubated at 4 °C overnight with monoclonal antibodies to the BKV major capsid protein VP1 (Santa Cruz Biotech) at 1:250 dilution. A synaptopodin antibody (Santa Cruz Biotech) was used at 1:250 dilution and the GAPDH antibody (Santa Cruz Biotech) was used at 1:3000 dilution. Membranes were washed five times in 0.1% TBST and incubated for one hour with a corresponding secondary antibody conjugated with HRP (ThermoFisher Scientific) at a dilution of 1:50,000. Immunoreactive bands were detected with WesternBright ECL (Advansta, San Jose, CA) following exposure to X-ray film. 

### 2.7. Statistical Analysis

Experiments presented in this study were performed independently three times under similar conditions. Data are presented as means with SDs. 

## 3. Results

### 3.1. BKV Lytic Replication in Human Glomerular Podocytes

**Figure 1:**
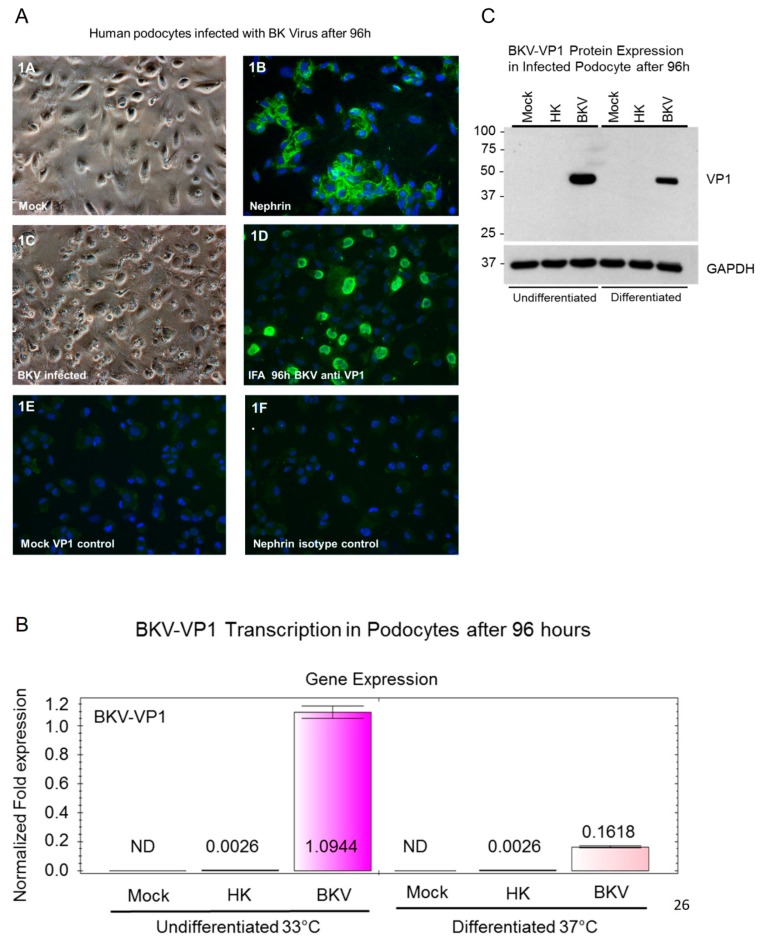
Immunofluorescence analysis of BKV replication in podocytes. (**A-1**) Phase-contrast images of mock-infected podocytes, (**A-2**) immunofluorescence staining of mock-infected glomerular podocytes stained with the nephrin antibody, (**A-3**) BKV-infected podocytes 96 h post-infection (**A-4**), immunofluorescence staining of BKV-infected podocytes with the BKV VP1 antibody 96 h post-infection (**A-5**), and mock-infected podocytes stained with the BKV VP1 antibody (**A-6**) mock-infected podocytes stained with the nephrin antibody. Nuclei were stained blue with 4′,6-diamidino-2-phenylindole (DAPI). All images were obtained using a Nikon TE2000S microscope mounted with a charge-coupled device (CCD) camera at ×200 magnification. (**B**) VP1 Expression in BKV-Infected podocytes. qRT-PCR analysis of VP1 transcripts in mock-infected podocytes or podocytes exposed to heat-killed (HK) BKV or infected with wild-type BKV for 96 h. qRT-PCR analysis was performed on both undifferentiated podocytes cultured at 33 °C and podocytes differentiated for 7 days at 37 °C. qRT-PCR results were normalized to GAPDH. (**C**) Western blot analysis of BKV VP1 protein expression in undifferentiated and differentiated podocytes mock-infected or exposed to heat-killed (HK) BKV or infected with wild-type BKV for 96 h. Protein size markers are shown in kilodaltons. GAPDH is used as a protein loading control.

**Figure 2:**
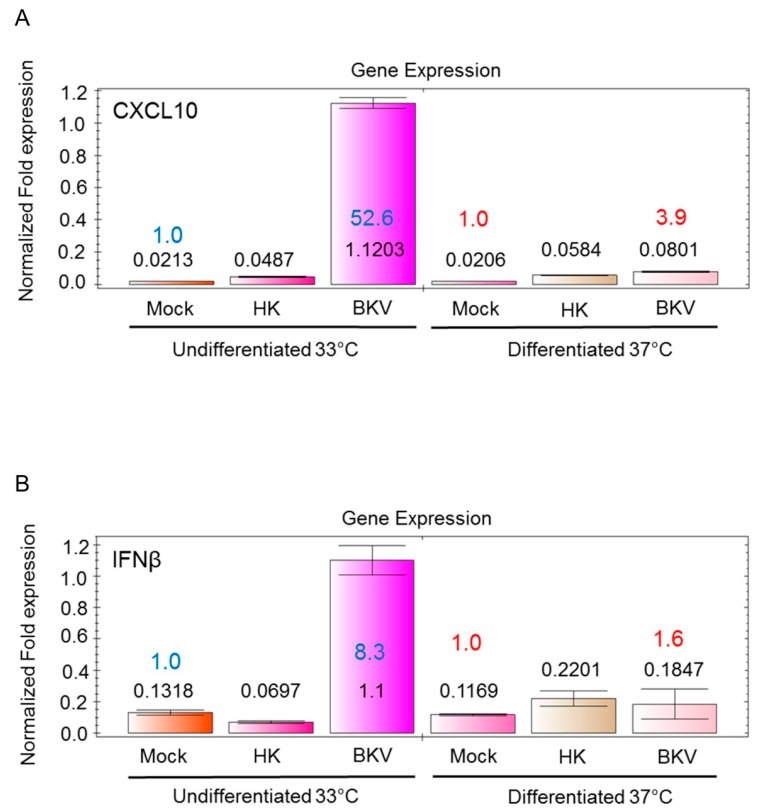
BKV induction of IFNβ and CXCL10 transcripts in podocytes. (**A**) qRT-PCR analysis of CXCL10 transcripts in mock-infected podocytes, podocytes exposed to heat-killed (HK) BKV, and podocytes infected with wild-type BKV for 96 h. qRT-PCR analysis was performed on undifferentiated podocytes culture at 33 °C and differentiated podocytes cultured at 37 °C for 7 days. qRT-PCR results were normalized to GAPDH. (**B**) qRT-PCR analysis of IFNβ expression in mock-infected podocytes and podocytes exposed to heat-killed (HK) or with wild-type BKV for 96 h. Results are shown for undifferentiated and differentiated podocytes. qRT-PCR results were normalized to GAPDH.

**Figure 3:**
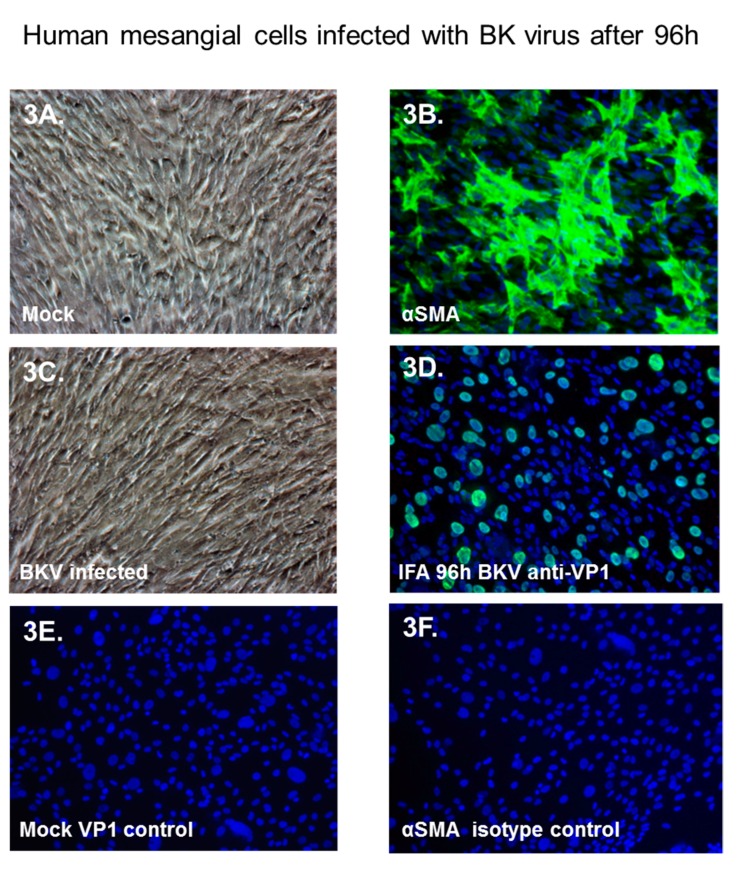
Immunofluorescence analysis of BKV replication in mesangial cells. (**A**), Phase-contrast image of mock-infected mesangial cells, (**B**) immunofluorescence staining of mock-infected mesangial cells stained with the α-smooth muscle actin (αSMA) antibody, (**C**) Phase-contrast image of BKV-infected mesangial cells 96 h after infection (**D**), immunofluorescence staining of BKV-infected mesangial cells with the SV40 Large-T-antigen (SV40-LTAg) antibody 96 h post-infection (**E**), and mock-infected mesangial cells stained with the SV40-LTAg antibody. (**F**) mock-infected mesangial cells stained with the αSMA antibody. Nuclei were stained blue with DAPI. All images ×200 magnification.

**Figure 4:**
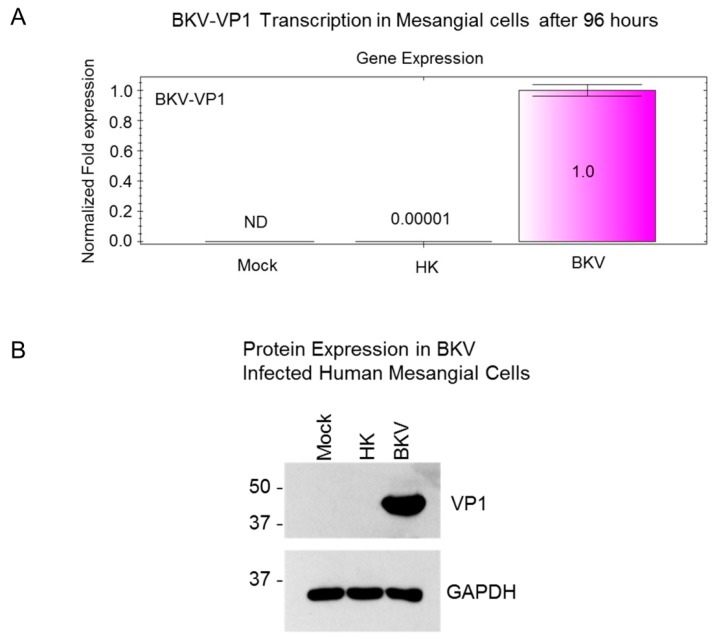
VP1 Expression in BKV-Infected mesangial cells. (**A**) qRT-PCR analysis of VP1 transcripts in mock-infected mesangial cells or cells exposed to heat-killed (HK) BKV or infected with wild-type BKV for 96 h. qRT-PCR results were normalized to GAPDH. (**B**) Western blot analysis of BKV VP1 expression in mesangial cells mock-infected or exposed to heat-killed or wild-type BKV for 96 h. Protein size markers are shown in kilodaltons. GAPDH is used as a protein loading control.

**Figure 5:**
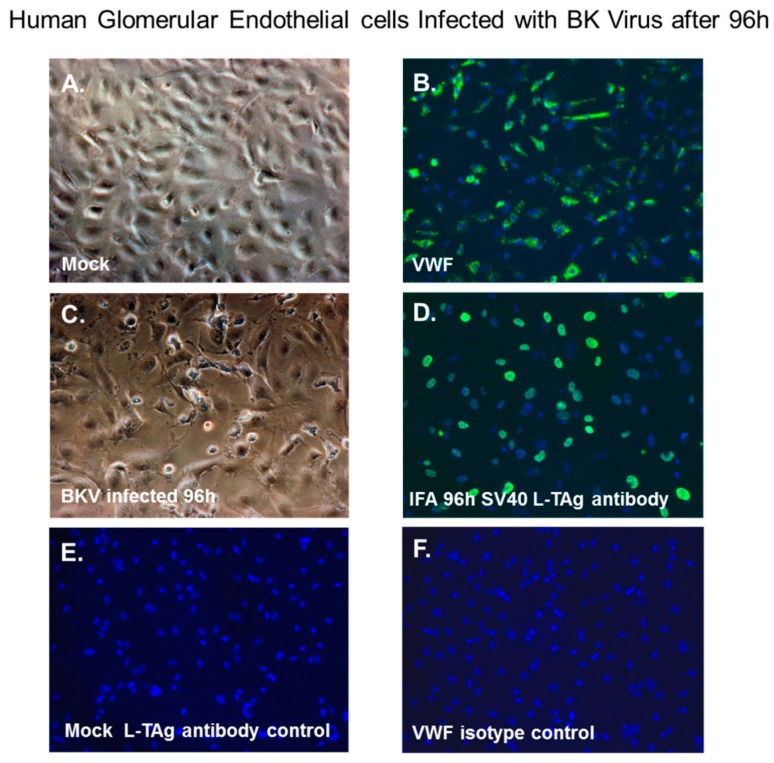
Immunofluorescence analysis of BKV replication in human glomerular endothelial cells. (**A**) Phase-contrast images of mock-infected glomerular endothelial cells, (**B**) immunofluorescence staining of mock-infected glomerular endothelial cells stained with the Von Willebrand factor (VWF) antibody, (**C**) BKV-infected glomerular endothelial cells 96 h after infection, (**D**) immunofluorescence staining of BKV-infected glomerular endothelial cells with the SV40 Large T-Antigen mouse monoclonal antibody 96 h post-infection, (**E**) mock-infected glomerular endothelial cells stained with the SV40 Large T-Antigen antibody, (**F**) mock-infected glomerular endothelial cells stained with the VWF isotype control antibody. Nuclei were stained blue with DAPI. All images ×200 magnification.

**Figure 6:**
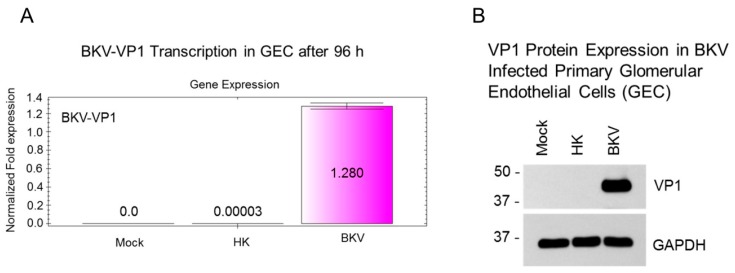
VP1 expression in BKV-Infected glomerular endothelial cells. (**A**) qRT-PCR analysis of mock-infected glomerular endothelial cells or cells exposed to heat-killed or wild-type BKV for 96 h. PCR results were normalized to GAPDH. (**B**) Western blot analysis of BKV VP1 protein expressed in glomerular endothelial cells exposed to mock, heat-killed BKV or wild-type BKV for 96 h. Protein size markers are in kilodaltons. GAPDH is used as a loading control.

BKV establishes a lifelong persistent infections in the kidneys, and viral shedding occurs in the urine. The effects of BKV on other glomerular parenchymal cells are unknown. We first examined established podocyte cultures and found podocytes to be morphologically typical of primary podocytes cell lines cultured in vitro (Figure 1A-1). These podocytes stained positive for the podocyte biomarker nephrin (Figure 1A-2). Next, podocytes were exposed to BKV at a multiplicity of infection of 0.1. At 96 h post-exposure, we observed BKV cytopathic effects that included rounding and sloughing of cells, as well as cytolysis (Figure 1A-3). Podocyte infection with BKV was confirmed by immunofluorescence staining with the BKV-VP1 antibody 96 h post-exposure (Figure 1A-4). Virus-infected podocytes showed nuclear staining with the BKV-VP1 antibody (Figure 1A-4). Controls for immunofluorescence staining included mock-infected podocytes stained with the BKV VP1 antibody (Figure 1A-5) and an isotype control for the nephrin antibody (Figure 1A-6). 

### 3.2. BKV Infected Undifferentiated Podocytes Express Higher Levels of VP1 Transcripts

BKV VP1 transcriptional analysis was performed by qRT-PCR in both undifferentiated podocytes cultivated at 33 °C and differentiated podocytes cultivated at 37 °C for 96 h post-infection (Figure 1B). We observed a robust 421-fold increase in the expression of VP1 transcripts in undifferentiated podocytes when compared with undifferentiated podocytes exposed to heat-killed BKV (Figure 1B). We observed a 62-fold increase in VP1 transcript levels in differentiated podocytes when compared with differentiated podocytes exposed to heat-killed BKV (Figure 1B). No detectable expression of VP1 transcripts was observed in either undifferentiated or differentiated mock infected control cells (Figure 1B). 

### 3.3. VP1 Protein Expression in Infected Undifferentiated and Differentiated Podocytes

VP1 total protein expression in undifferentiated and differentiated podocytes was confirmed by Western blot analysis (Figure 1C). The highest level of VP1 protein expression was observed in undifferentiated podocytes exposed to BKV when compared with differentiated podocytes (Figure 1C). No detectable expression of VP1 protein was observed in mock-infected podocytes or podocytes exposed to heat-killed virus (Figure 1C). 

### 3.4. BKV Infection Induces Higher IFNβ and CXCL10 Gene Expression in Undifferentiated Podocytes

The activation of the innate immune defense mechanisms during BKVAN is poorly understood. The proinflammatory cytokine CXCL10/IP-10 is known to stimulate the migration and activation of immune effector cells to sites of infection [31]. BKV induces inflammation via the up-regulation of CXCL10 protein expression in renal transplant patients with BKVAN [31]. We examined whether BKV infection stimulates CXCL10 transcription in undifferentiated and differentiated podocytes using qRT-PCR. We observed an over 52-fold increase in CXCL10 gene transcription in undifferentiated podocytes compared with mock infected undifferentiated podocytes after 96 h, respectively (Figure 2A). A moderate, about 4-fold increase occurred in the expression of CXCL10 transcripts in differentiated podocytes compared to mock-infected (Figure 2A). Overall, we observed a more robust induction of CXCL10 gene expression in undifferentiated podocytes compared to differentiated podocytes (Figure 2A). In undifferentiated BKV-infected podocytes, we observed an over 8-fold increase in IFNβ gene transcription as compared with mock-infected control cells (Figure 2B). In BKV-infected differentiated podocytes, only a marginal 1.6-fold increase in IFNβ gene expression occurred as compared to mock-infected cells (Figure 2B). 

### 3.5. BKV Replication in Human Primary Renal Mesangial Cells

Next, we examined BKV infection of primary renal mesangial cells. Normal human mesangial cells appeared typical as described by the manufacturer (ScienCell) (Figure 3A) and stained positive for a mesangial cell biomarker, α-smooth muscle actin (α-SMA, Figure 3B). Mesangial cells exposed to BKV for 96 h showed no clear evidence of BKV-associated cytopathological changes (Figure 3C). Despite this, we observed high levels of virus replication, as demonstrated by nuclear staining of BKV-infected mesangial cells with the SV40 Large T-Ag antibody (Figure 3D). No significant background staining was observed in mock-infected mesangial cells stained with the SV40 Large T-Ag antibody (Figure 3E) or mock-infected mesangial cells stained with an α-SMA IgG isotype antibody (Figure 3F). 

To examine whether the lack of cytopathic effects in infected mesangial cells is due to inefficient BKV replication in these cells, we analyzed the expression of BKV VP1 transcripts and VP1 protein (Figure 4A). BKV VP1 transcription analysis was performed by qRT-PCR in mesangial cells 96 h post-infection (Figure 4A). We observed a 100,000-fold increase in VP1 transcription in BKV-infected mesangial cells when compared with mesangial cells exposed to heat-killed BKV (Figure 4A). No detectable VP1 transcription was observed in mock-infected control cells (Figure 4A). Accordingly, VP1 protein expression was observed only in BKV-infected mesangial cells. No detectable expression of VP1 protein was observed in mock-infected mesangial cells or mesangial cells exposed to heat-killed virus (Figure 4B).

### 3.6. BKV Lytic Replication in Human Glomerular Endothelial Cells

Next, we examined renal glomerular endothelial cells for BKV infectivity. Normal glomerular endothelial cells exhibited a characteristic cobblestone-like morphology (Figure 5A) and stained positive for the endothelial cell biomarker von Willebrand factor (VWF, Figure 5B). At 96 h after BKV exposure, these cells showed evidence of cytopathology by phase microscopy (Figure 5C) and showed nuclear staining with the SV40 Large T-Ag antibody (Figure 5D). Controls for immunofluorescence staining included mock-infected glomerular endothelial cells stained with the SV40 Large T-Ag antibody (Figure 5E) and an isotype control for the von Willebrand factor antibody (Figure 5F). 

### 3.7. BKV Infected Glomerular Endothelial Cells Express High Levels of VP1 Transcripts and VP1 Protein

To confirm that the observed cytopathic morphology of glomerular endothelial cells is related to BKV infection, we analyzed the expression of BKV VP1 transcripts and protein (Figure 6A). BKV-VP1 transcription analysis was performed at 96 h post infection (Figure 6A). We observed a 42,667-fold increase in VP1 transcription in BKV infected glomerular endothelial cells when compared to glomerular endothelial cells exposed heat-killed BKV (Figure 6A). No detectable VP1 transcription was observed in mock infected control cells (Figure 6A). We then examined the expression of VP1 protein by western blot analysis (Figure 6B). As expected, VP1 protein expression was detected only in BKV infected glomerular endothelial cells (Figure 6B).

### 3.8. BKV-Induced VP1 Transcription Increases Over Time in BKV Infected GVU Cells

**Figure 7:**
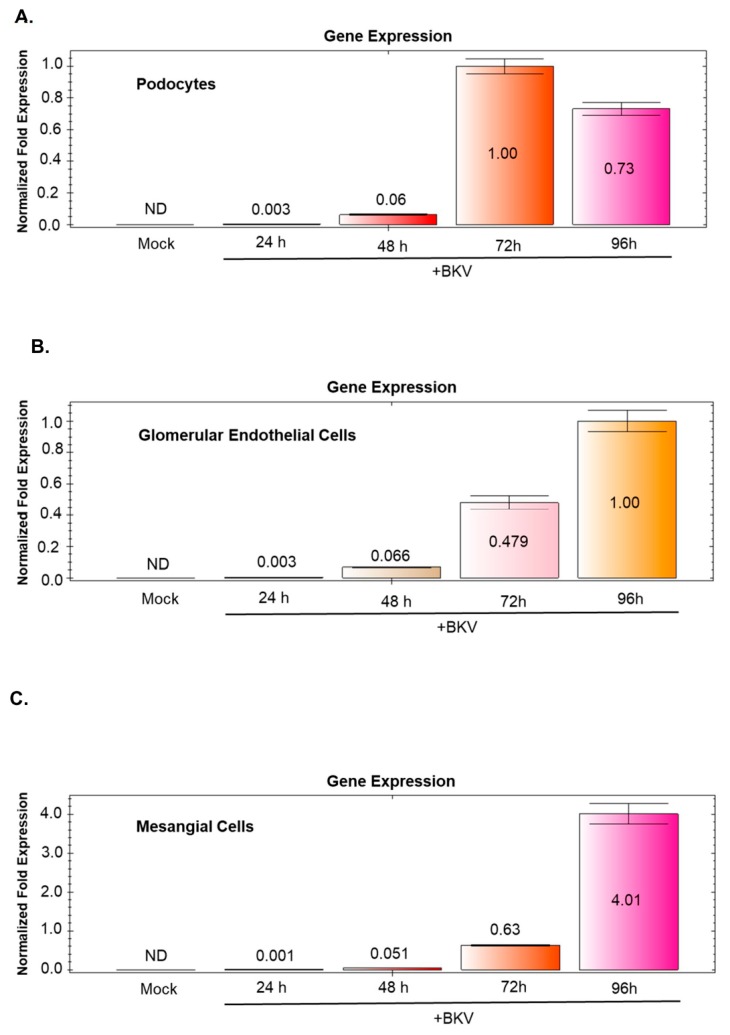
Time course analysis of VP1 expression in BKV-Infected GVU cells. (**A**) qRT-PCR analysis of mock-infected undifferentiated podocytes and podocytes infected with BKV for 24, 48, 72, and 96 h. (**B**) qRT-PCR analysis of mock-infected glomerular endothelial cells and glomerular endothelial cells infected with BKV for 24, 48, 72, and 96 h. (**C**) qRT-PCR analysis of mock-infected mesangial cells and mesangial cells infected with BKV for 24, 48, 72, and 96 h. PCR results were normalized to GAPDH.

Time course infection analysis of BKV infected GVU cells shows an increase in BKV VP1 transcription when compared to mock infected controls (Figure 7A–C). Glomerular endothelial cells and mesangial cells show a consistent increase in BKV VP1 gene expression at 24, 48, 72 and 96 h post-infection (Figure 7B,C). Podocytes show a consistent increase in BKV VP1 gene expression at 24, 48 and 72 h post-infection (Figure 7A). However, we observed a 23% decrease in BKV VP1 gene expression from 72 to 96 h post infection (Figure 7A). 

### 3.9. BKV Infection of Podocytes and Mesangial Cells Induces CXCL10 and IFNβ Gene Expression that Correlates with Increased Virus Replication

**Figure 8:**
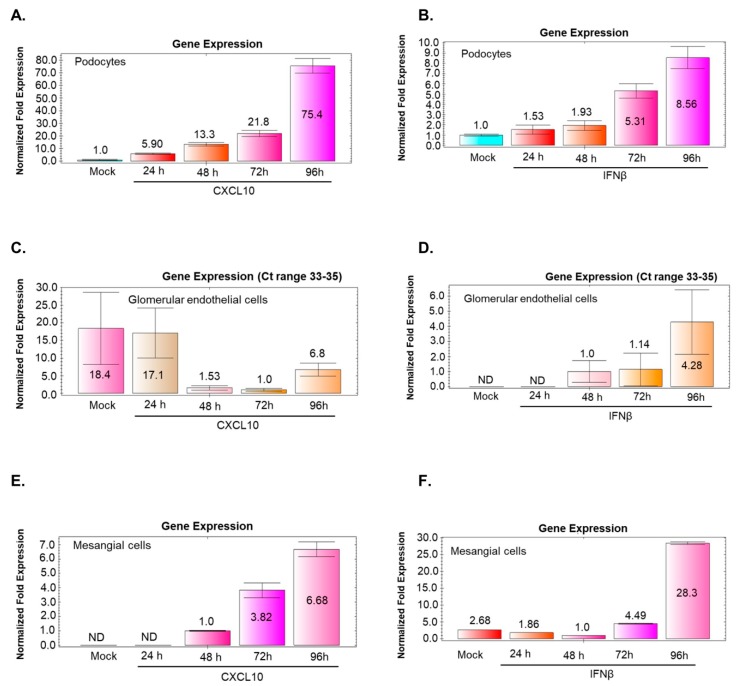
Time course analysis of CXCL10 and IFNβ gene expression in BKV-Infected GVU cells. (**A**,**B**) qRT-PCR analysis CXCL10 and IFNβ gene expression in mock-infected undifferentiated podocytes and podocytes infected with BKV for 24, 48, 72, and 96 h, respectively. (**C**,**D**) qRT-PCR analysis CXCL10 and IFNβ in mock-infected glomerular endothelia cells and glomerular endothelial cells infected with BKV for 24, 48, 72, and 96 h, respectively. Note that Ct values for CXCL10 and IFNβ gene expression are in the range of 33–35, below statistical significance (**E**,**F**). qRT-PCR analysis CXCL10 and IFNβ in mock-infected mesangial cells and mesangial cells infected with BKV for 24, 48, 72, and 96 h respectively. PCR results were normalized to GAPDH.

Time course analysis for CXCL10 and IFNβ gene expression was examined in mock and BKV-infected GVU cells. qRT-PCR analysis of CXCL10 gene expression in mock-infected podocytes and podocytes infected with BKV showed an incremental induction of CXCL10 of 5.9, 13.3, 21.8, and 75.4-fold after 24, 48, 72, and 96 h, respectively (Figure 8A). Similarly, undifferentiated podocytes infected with BKV showed an incremental induction of IFNβ of 1.53, 1.93, 5.31, and 8.56-fold after 24, 48, 72, and 96 h, respectively (Figure 8B). Mesangial cells infected with BKV showed an incremental induction of CXCL10 gene expression, which picks (6.7-fold) at 96 h (Figure 8E) and a more significant induction of IFNβ gene expression (28.3-fold) observed at 96 h post infection (Figure 8F).

### 3.10. BKV Infection of Glomerular Endothelial Cells does not Noticeably Stimulate CXCL10 or IFNβ Gene Expression

Time course analysis shows negligible changes in CXCL10 and IFNβ gene expression in glomerular endothelial cells infected with BKV (Figure 8C,D). The respective C_t_ values for both transcripts that accumulated in mock and BKV-infected cells during 24–96 h were in the range of 33–35, (Figure 8C,D) suggesting that efficient BKV replication in these cells (Figure 6A and Figure 7B) does not induce CXCL10 and IFNβ gene expression.

### 3.11. Hypothetical Model for BKV Dissemination in Glomerulus.

**Figure 9:**
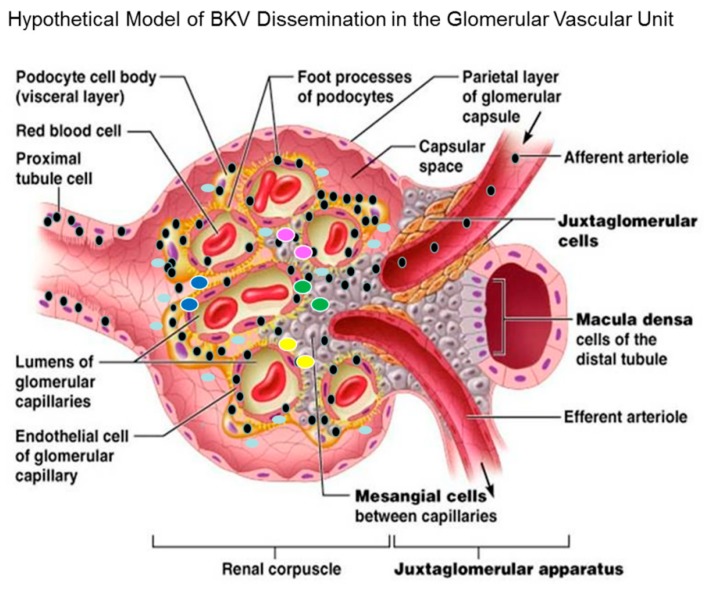
BK virus (black spheres) enters the glomerulus of the renal compartment via the afferent arteriole during the viremic phase leading to infection of mesangial cells. The virus spreads from mesangial cells to glomerular podocytes which are highly permissive for BK virus infection. Glomerular endothelial cells are also highly permissive for infection. Finally, the virus further disseminates and infect the proximal tubular epithelial cells. Lytic replication in GVU targets cells along with tubular epithelial cells contributes to viruria and inflammation. These cells likely serve as virus amplification reservoirs in the glomerulus upon BKV reactivation after immunosuppression. Model of BKV entry and existence in the glomerulus (modified with permission from Pearson Education Inc. 2013 [unpublished data]).

In the model, we proposed that the virus enters the bloodstream via the afferent arteriole and glomerular capillaries, leading to an infection of the renal corpuscle and subsequently the glomerular endothelial cells of the kidney (Figure 9). The virus spreads from infected glomerular endothelial cells to the glomerular parenchyma. Mesangial cells and podocytes are highly exposed to BKV. Podocytes, mesangial cells and proximal tubular epithelial cells are also highly permissive for BKV infection (Figure 9). Viremia with prolonged viral shedding ensues via the voided urine of infected patients. These findings suggests that GVU cells together could serve as amplification reservoirs in the kidney, resulting in high-level persistent viremia and viruria, as measured by qRT-PCR for BKV RNA and decoy cells in urine. The current findings also show that the cytokine CXCL10 gene expression is strongly induced in podocytes and to a lesser extent in mesangial cells, which could indicate enhanced migration of immune cells to sites of virus infection that could lead to increased inflammation observed in BKVAN. CXCL10 expression (aqua ovals) by podocytes and mesangial cells could serve as a chemoattractant for monocytes/macrophages (yellow ovals), T cells (green ovals), NK cells (blue ovals) and dendritic cells (pink ovals) and promote T cell adhesion to endothelial cells (Figure 9). The recruitment of immune effector cells to sites of BKV lytic replication in GVU cells could lead to early events in the development of BKVAN (Figure 9).

## 4. Discussion

The investigation of mechanisms of BKV pathogenesis has primarily centered on proximal tubular epithelial cells as both the reservoir and perpetrator of pathogenic pathways leading to fibrosis associated with BKVAN. However, a comprehensive study of glomerular cells infectivity for BKV has not been performed and requires further investigation. Here, we examined human renal glomerular parenchymal cells for BKV infection and observed high-level viral infectivity and replication in podocytes, mesangial cells, and glomerular endothelial cells (GVU). However, we observed cytopathology in BKV-infected podocytes and glomerular endothelial cells but not mesangial cells. We also observed a differential infection of differentiated podocytes compared to undifferentiated podocytes. We speculate that this could be due to differential receptor expression, down regulation of the primary receptor, or induction of an antiviral host factor(s). Previous studies provided evidence of BKV infection of GVU cells by ultrastructural observations of kidney biopsy tissue in a case of BKV nephropathy, with the virus detected in glomerular subepithelial humps [32]. They also provide evidence of cytoplasmic clearance of viruses from the glomerular basement membrane by podocytes [32]. However, no definitive evidence of direct infection of podocytes was provided. Lee et al., observed tubulo-reticular inclusions in glomerular endothelial cells and peritubular capillaries by ultrastructural analysis in kidney tissue from patients with BKVAN; however, no evidence of infection of glomerular endothelial cells was observed [33]. We are also aware that BKV is rarely found in the glomerulus of biopsy specimens from patients with BKVAN. However, we show evidence of GVU cell infection and cytopathology in human primary cell cultures as well BKV late gene and protein expression in GVU. We show an increase in BKV VP1 transcription in a time course analysis. These findings suggest that although infection of these GVU cells may be uncommon in vivo, it may represent early events in the pathogenesis of BKVAN.

IFNβ and CXCL10 were shown to be induced in renal tissue from patients with BKVAN [34]. Jia et al., analyzed public array data by bioinformatics and have identified key protein interaction networks of BKV nephropathy in patients receiving kidney transplantations [35]. They proposed, that CXCL10, EGF, and STAT1 may induce renal injury by promoting inflammation and inhibiting the repair of tissue damage in patients with BKVAN [35]. Increased levels of CXCL10 were found in the serum and renal tissue of patients with BKVAN when compared to patients with non-rejection allografts [18,36]. They also observed upregulation of CXCL10 at translation levels in renal transplant patients with nephropathy [18]. Jackson et al., showed that urine CXCL9 and CXCL10 were markedly elevated in adults and children experiencing either acute rejection when compared to stable allograft recipients [37]. They also reported that stimulation of inflammation via CXCL10 could contributes to renal graft loss in patients with BKVAN [18]. Hirt-Minkowski et al. showed that urinary CXCL10 had clinically useful diagnostic properties for detection of clinical and subclinical tubulointerstitial inflammation in transplant patients [38,39].

We observed a strong transcriptional induction of the pro-inflammatory cytokine CXCL10 and lower levels of IFNβ in BKV-infected undifferentiated podocytes sustained over 96 h post-infection. Interestingly, BKV infected differentiated podocytes showed a significantly smaller increase of CXCL10 and a marginal increase of IFNβ transcripts and at 96 h post-infection, as compared with undifferentiated podocytes. In a time course analysis of BKV-Infected GVU cells, we observed in mesangial cells a significant induction of IFNβ gene and somewhat lower induction of CXCL10 gene expression. In contrast, no significant changes in CXCL10 and IFNβ gene expression were observed in BKV-infected glomerular endothelial cells, despite that BKV replicated efficiently in these cells. This could possibly be due to the shutoff of CXCL10 and IFNβ gene expression by replicating BKV in these cells. Renal proximal tubular epithelial cells have been identified as BKV target cells for latency and reactivation in vivo. Our observation supports the notion that GVU cells may possibly have a role as potential latency reservoirs in vivo for virus amplification in BKVAN and may represent additional target cells for BKV reactivation after immunosuppression.

## 5. Conclusions

Cellular components of the human renal GVU are permissive for BKV infectivity in vitro. BKV replication in GVU cells could lead to glomerular cell injury, cytolysis and inflammation associated with BKVAN. Taken together, these findings suggest that GVU cells may represent potential newly identified sites for viral latency and sites of amplification reservoirs upon reactivation via immunosuppression. BKV may induce CXCL10 in podocytes and mesangial cells that would aid in the recruitment of immune effector cells, enhance glomerular inflammation and could contribute to fibrosis in BKVAN. The next steps for these studies will be the in vivo validation of BKV infectivity for GVU cells in glomerular tissue from renal transplant patients with BKVAN.

## References

[1] Gardner S.D., Field A.M., Coleman D.V., Hulme B. (1971). New human papovavirus (B.K.) isolated from urine after renal transplantation. Lancet.

[2] Shah K.V., Fields B.N., Knipe K.M., Howley P.M. (1990). Polyomaviruses. Virology.

[3] Hirsch H.H. (2005). BK virus: Opportunity makes a pathogen. Clin. Infect. Dis..

[4] Ahsan N., Shah K.V. (2006). Polyomaviruses and Human Diseases.

[5] Siguier M., Sellier P., Bergmann J.-F. (2012). BK-virus infections: A literature review. Med. Mal. Infect..

[6] Nickeleit V., Singh H.K. (2015). Polyomaviruses and disease: Is there more to know than viremia and viruria?. Curr. Opin. Organ Transplant..

[7] Teutsch K., Schweitzer F., Knops E., Kaiser R., Pfister H., Verheyen J., Göbel H., Clingöz T., Di Cristanziano V. (2015). Early identification of renal transplant recipients with high risk of polyomavirus-associated nephropathy. Med. Microbiol. Immunol..

[8] Bennett J.E., Dolin R., Blaser M.J. (2015). Mandell, Douglas, and Bennett’s Principles and Practice of Infectious Diseases.

[9] Pinto M., Dobson S. (2014). BK and JC virus: A review. J. Infect..

[10] Vago L., Cinque P., Sala E., Nebuloni M., Caldarelli R., Racca S., Ferrante P., Trabattoni G.R., Costanzi G. (1996). JCV-DNA and BKV-DNA in the CNS tissue and CSF of AIDS patients and normal subjects. Study of 41 cases and review of the literature. J. Acquir. Immune Defic. Syndr. Hum. Retrovirol..

[11] Lesprit P., Chaline-Lehmann D., Authier F.-J., Ponnelle T., Gray F., Levy Y. (2001). BK virus encephalitis in a patient with AIDS and lymphoma. AIDS.

[12] Jiang M., Abend J.R., Johnson S.F., Imperiale M.J. (2009). The role of polyomaviruses in human disease. Virology.

[13] Hirsch H.H., Randhawa P. (2013). AST Infectious Diseases Community of Practice. BK polyomavirus in solid organ transplantation. Am. J. Transplant..

[14] Dalianis T., Hirsch H.H. (2013). Human polyomaviruses in disease and cancer. Virology.

[15] Binet I., Nickeleit V., Hirsch H.H., Prince O., Dalquen P., Gudat F., Mihatsch M.J., Thiel G. (1999). Polyomavirus disease under new immunosuppressive drugs: A cause of renal graft dysfunction and graft loss. Transplantation.

[16] Comoli P., Binggeli S., Ginevri F., Hirsch H.H. (2006). Polyomavirus-associated nephropathy: Update on BK virus-specific immunity. Transplant. Infect. Dis..

[17] Han S.B., Cho B., Kang J.H. (2014). BK virus-associated hemorrhagic cystitis after pediatric stem cell transplantation. Korean J. Pediatr..

[18] Kariminik A., Dabiri S., Yaghobi R. (2016). Polyomavirus BK induces inflammation via up-regulation of CXCL10 at translation levels in renal transplant patients with nephropathy. Inflammation.

[19] Egli A., Binggeli S., Bodaghi S., Dumoulin A., Funk G.A., Khanna N., Leuenberger D., Gosert R., Hirsch H.H. (2007). Cytomegalovirus and polyomavirus BK posttransplant. Nephrol. Dial. Transplant..

[20] Burrows N.R., Li Y., Williams D.E. (2008). Racial and ethnic differences in trends of end-stage renal disease: United States, 1995 to 2005. Adv. Chronic Kidney Dis..

[21] Palmer Alves T., Lewis J. (2010). Racial differences in chronic kidney disease (CKD) and end-stage renal disease (ESRD) in the United States: A social and economic dilemma. Clin. Nephrol..

[22] Regunathan-Shenk R., Hussain F.N., Ganda A. (2016). Chronic kidney disease and end-stage renal disease in disadvantaged communities of North America: An investigational challenge to limit disease progression and cardiovascular risk. Clin. Nephrol..

[23] Albertus P., Morgenstern H., Robinson B., Saran R. (2016). Risk of ESRD in the United States. Am. J. Kidney Dis..

[24] Alcendor D.J. (2017). Zika Virus Infection of the Human Glomerular Cells: Implications for Viral Reservoirs and Renal Pathogenesis. J. Infect. Dis..

[25] Popik W., Correa H., Khatua A., Aronoff D.M., Alcendor D.J. (2018). Mesangial cells, specialized renal Pericytes, and cytomegalovirus infectivity: Implications for HCMV pathology in the glomerular vascular unit and post-transplant renal disease. J. Transl. Sci..

[26] Saleem M.A., O’Hare M.J., Reiser J. (2002). A conditionally immortalized human podocyte cell line demonstrating nephrin and podocin expression. J. Am. Soc. Nephrol..

[27] Khatua A.K., Taylor H.E., Hildreth J.E.K., Popik W. (2010). Non-productive HIV-1 infection of human glomerular and urinary podocytes. Virology.

[28] Seif I., Khoury G., Dhar R. (1979). The genome of human papovavirus BKV. Cell.

[29] Takemoto K.K., Mullarkey M.F. (1973). Human papovavirus, BK strain: Biological studies including antigenic relationship to simian virus 40. J. Virol..

[30] Wilkerson I., Laban J., Mitchell J.M., Sheibani N., Alcendor D.J. (2015). Retinal pericytes and cytomegalovirus infectivity: Implications for HCMV-induced retinopathy and congenital ocular disease. J. Neuroinflamm..

[31] Dabiri S., Kariminik A., Kennedy D. (2016). The role of CXCR3 and its ligands in renal transplant outcome. Eur. Cytokine Netw..

[32] Brealey J.K. (2007). Ultrastructural observations in a case of BK virus nephropathy with viruses in glomerular subepithelial humps. Ultrastruct. Pathol..

[33] Lee J.Y., Song S.H., Kim Y.S., Lim B.J., Kim S.I., Kim M.S., Jeong H.J. (2017). Tubuloreticular inclusions in peritubular capillaries of renal allografts. Pathol. Res. Pract..

[34] Assetta B., De Cecco M., O’Hara B., Altwood W.J. (2016). JC polyomavirus infection of primary human renal epithelial cells is controlled by a type I IFN-induced response. MBio.

[35] Jia L., Fu W., Jia R. (2018). Identification of potential key protein interaction networks of BK virus nephropathy in patients receiving kidney transplantation. Sci. Rep..

[36] Hu H., Aizenstein B.D., Puchalski A., Burmania J.A., Hamawy M.M., Knechtle S.J. (2004). Elevation of CXCR3-binding chemokines in urine indicates acute renal-allograft dysfunction. Am. J. Transplant..

[37] Jackson J.A., Kim E.J., Begley B., Cheeseman J., Harden T., Perez S.D., Thomas S., Warshaw B., Kirk A.D. (2011). Urinary chemokines CXCL9 and CXCL10 are noninvasive markers of renal allograft rejection and BK viral infection. Am. J. Transplant..

[38] Hirt-Minkowski P., Amico P., Ho J., Gao A., Bestland J., Hopfer H., Steiger J., Dickenmann M., Burkhalter F., Rush D. (2012). Detection of clinical and subclinical tubulo-interstitial inflammation by the urinary CXCL10 chemokine in a real-life setting. Am. J. Transplant..

[39] Schaub S., Nickerson P., Rush D., Mayr M., Hess C., Golian M., Stefura W., HayGlass K. (2009). Urinary CXCL9 and CXCL10 levels correlate with the extent of subclinical tubulitis. Am. J. Transplant..

